# A dynamically reconfigurable Fano metamaterial through graphene tuning for switching and sensing applications

**DOI:** 10.1038/srep02105

**Published:** 2013-07-01

**Authors:** M. Amin, M. Farhat, H. Baǧcı

**Affiliations:** 1 Division of Computer, Electrical, and Mathematical Sciences and Engineering, King Abdullah University of Science and Technology (KAUST), Thuwal, 23955-6900, Saudi Arabia

## Abstract

We report on a novel electrically tunable hybrid graphene-gold Fano resonator. The proposed metamaterial consists of a square graphene patch and a square gold frame. The destructive interference between the narrow- and broadband dipolar surface plasmons, which are induced respectively on the surfaces of the graphene patch and the gold frame, leads to the plasmonic equivalent of electromagnetically induced transparency (EIT). The response of the metamaterial is polarization independent due to the symmetry of the structure and its spectral features are shown to be highly controllable by changing a gate voltage applied to the graphene patch. Additionally, effective group index of the device is retrieved and is found to be very high within the EIT window suggesting its potential use in slow light applications. Potential outcomes such as high sensing ability and switching at terahertz frequencies are demonstrated through numerical simulations with realistic parameters.

The idea of electromagnetically induced transparency (EIT) and the concept of Fano resonance were originally discovered in the context of quantum mechanics^1^. EIT is generated when a narrowband discrete state destructively interferes with a broadband continuum; the resulting spectrum has the Fano line-shape^1^. Plasmonic analogues of EIT have been recently generated via coupling either antiparallel dipolar surface plasmon polaritons (SPPs) or bright dipolar SPPs with dark higher order ones^2^,^3^, all induced on metal-dielectric interfaces. The excitation of the originally dark higher order SPPs is often made possible by means of symmetry breaking^3^,^4^,^5^. Generation of physical phenomena equivalent to EIT at terahertz (THz) frequencies is currently one of the most exciting topics of plasmonics research^2^. Since most of the biomolecules have their vibrational modes oscillating at THz frequencies^6^,^7^, this means that EIT generated at these frequencies offers a viable way for biosensing. The drastic “slowing” of light around the narrow Fano resonance results in an increased sensitivity to changes in the medium's refractive index. Plasmonic EIT could also be used in designing efficient switches for modulating the amplitude and phase of waves transmitted through metamaterials^8^. Possibility of designing ultra-sensitive bio-detectors and efficient switches urges the investigation of plasmonic metamaterials capable of supporting Fano resonances at THz frequencies. The unique and highly tunable electrical properties of graphene^9^ observed within this band of the spectrum render it an attractive candidate as a building block of such metamaterials.

Since its first practical isolation by Novoselov and Geim in 2004^9^, graphene solicited a keen interest among physicists and engineers. This could be attributed to graphene's unprecedented properties that cannot be found in any other material^10^. High electron mobility^11^, significant white light absorption^8^,^12^,^13^, ability to support SPPs^14^,^15^,^16^,^17^ are among these characteristics that are relevant in the fields of electromagnetics, optics, and photonics^18^.

In this work, graphene's ability to support SPPs at THz frequencies is exploited to design a Fano resonator that hybridizes SPPs generated on graphene and gold surfaces. Graphene surface plasmon polaritons (GSPPs) have several advantages when compared to SPPs generated on metallic surfaces: GSPPs have higher volume confinement (exceeding 10^6^ times the diffraction limit), are easier to tune (obtained via applying a gate voltage to the graphene), and propagate longer distances and have narrower spectral support (due to the lower intrinsic losses in graphene)^19^,^20^. Not surprisingly, these superior features of GSPPs have fueled research in several directions such as development of GSPP waveguides^21^,^22^, THz antennas^23^,^24^,^25^, perfect absorbers^26^,^27^, novel Fourier optics devices^28^, THz cloaks^29^,^30^, photonic crystal nano-cavities^31^, modulators^32^ and sensors^33^.

The hybrid graphene-gold Fano resonator proposed in this work is a doubly periodic array of a unit cell constructed using a square graphene patch located at the center of a square gold frame. The Fano resonance is obtained from the destructive interference between the dipolar SPPs generated on the graphene patch and the gold frame at THz frequencies and its spectral shape and location can easily be tuned by applying a gate voltage to the graphene patch.

## Results

### Physical mechanism to generate Fano resonance

**Figure 1:**
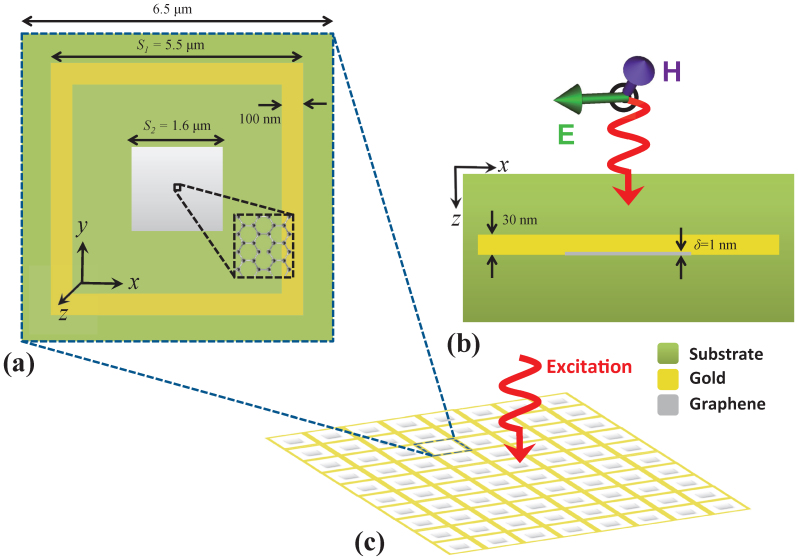
(a) Top view of the unit cell with dimensions.(b) Cross section view and the normally incident excitation. (c) Doubly periodic array of the unit cell and the normally incident excitation.

The Fano resonator proposed in this work is a doubly periodic array of a unit cell that consists of a square graphene patch located at the center of a square gold frame. The resonator is embedded in a dielectric substrate. (see Fig. 1). The proposed Fano resonator is excited with a plane wave propagating in the *z*-direction with electric field polarized in the *x*-direction [Figs. 1(b) and 1(c)]. It should be noted here that due to the symmetry of the unit cell and the same periodicity in *x*- and *y*- directions, the response of the resonator is polarization independent. When excited with this incident field separately, the gold frame and the graphene patch support dipolar SPPs. As a result, the resonator constructed using the gold frame and the graphene patch supports an SPP mode hybridized between these two dipolar modes. Since the dipolar SPP induced on the frame has a much broader spectral support than the one induced on the graphene patch due to the presence of higher intrinsic losses in gold, these two modes' destructive interference generates a Fano line-shape in the response of the resonator. It should be noted here that the plasma frequencies of gold and graphene are separated by a large offset. This means that the resonance frequencies corresponding to the dipolar SPPs induced on the gold frame and the graphene patch are expected to be separated by a large gap. To move the resonance frequencies closer to each other and have the SPPs' spectral supports overlap at THz frequencies, the geometrical dimensions of the frame and the patch are chosen to be in μm scale as shown in Fig. 1(a).

Obviously, in this design, SPP of the graphene patch acts like the “dark mode” of the traditional Fano resonator designs made of only metals^3^. Additionally, unlike these traditional resonators, the proposed design does not require its symmetry to be “broken” since the narrower mode can be directly excited by the incident field even if the structure is fully symmetric. The spectral location and line-shape of the graphene's narrow dipolar SPP is determined by graphene's complex relative permittivity *ε*_V,G_, which can be controlled by adjusting the chemical potential *μ_c_* (see Methods Section on Material Models for details). Variation in *μ_c_* can be achieved by applying a gate voltage to the graphene patch using nearly transparent electrodes without perturbing the response of the resonator as suggested recently in reference^8^.

### Proof of concept via numerical experiments

**Figure 2:**
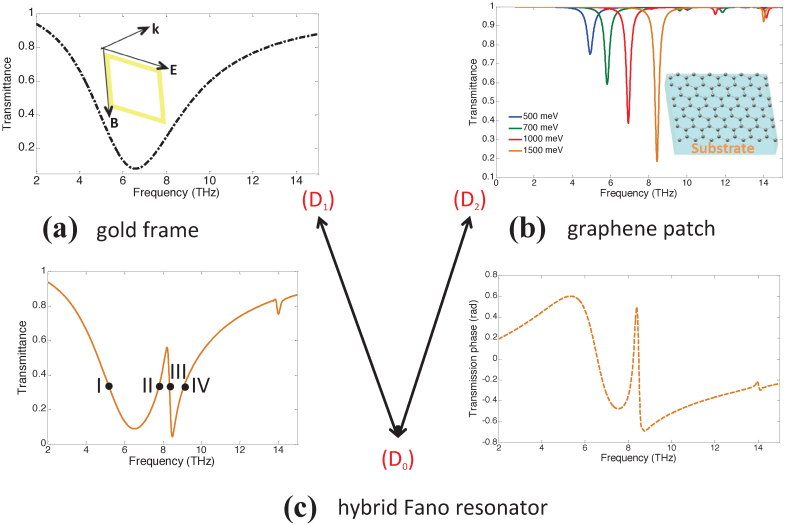
(a) Transmittance of only the gold frame.*S*_1_ = 5.5 μm and *ε_d_* = 3.5. SPP is marked as *D*_1_. (b) Transmittance of only the graphene patch for different values of *μ_c_*. *S*_2_ = 1.6 μm, *μ* = 10,000 cm^2^/Vs, and *ε_d_* = 3.5. SPP is marked as SPP *D*_2_. (c) Transmittance of the resonator with both the gold frame and the graphene patch. *S*_1_ = 5.5 μm, *S*_2_ = 1.6 μm, *ε_d_* = 3.5, *μ* = 10,000 cm^2^/Vs, and *μ_c_* = 1500 meV. Amplitude on the left-side and phase on the right-side.

The hybridization of the dipolar SPPs induced on the gold frame and the graphene patch is demonstrated by an example. For this simulation, the dimension of the gold frame *S*_1_ = 5.5 μm, the dimension of the graphene patch *S*_2_ = 1.6 μm, the relative permittivity of the substrate *ε_d_* = 3.5, and graphene's electron mobility *μ* = 10,000 cm^2^/Vs and chemical potential *μ_c_* = 1500 meV. It should be noted here that the value assigned to *μ* is a rather conservative choice considering the latest experimental results^18^. The transmittance of three structures constructed using unit cells with only the gold frame, only the graphene patch, and both the gold frame and the graphene patch are computed [Figs. 2(a), 2(b), and 2(c), respectively]. As expected, the dipolar SPPs induced on the gold frame (marked as *D*_1_) and the graphene patch (marked as *D*_2_) have broad (continuum-like) and very narrow spectral supports, respectively. Figure 2(c) clearly demonstrates the asymmetric Fano-like spectral line-shape and a narrow EIT window in the response of the resonator constructed using both the gold frame and the graphene patch, which results from destructive interference of *D*_1_ and *D*_2_.

**Figure 3:**
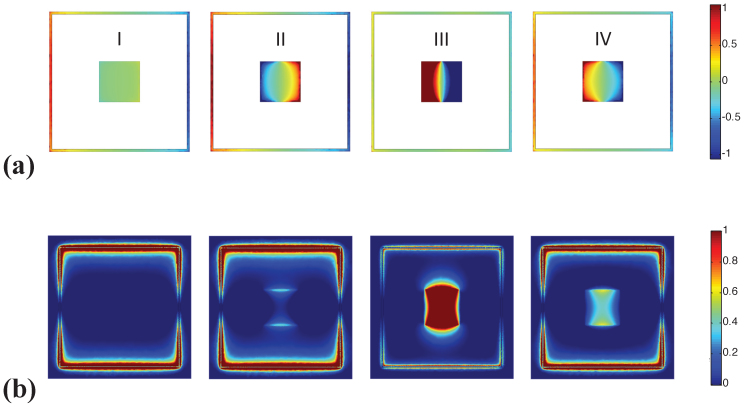
(a) Surface charge distributions on the unit cell computed at frequency points I, II, III, and IV corresponding to the frequencies: 5.3, 7.7, 8.35 and 9 THz.*S*_1_ = 5.5 μm, *S*_2_ = 1.6 μm, *ε_d_* = 3.5, *μ* = 10,000 cm^2^/Vs, and *μ_c_* = 1500 meV. The color scale for the positive and negative surface charges are normalized between (−1 and 1). (b) Same as in (a) but for the distribution of the norm of the magnetic field in the plane of the unit cell *x-y*.

The dipolar nature of the SPPs is exhibited in Fig. 3 showing the surface charge distributions and the magnetic field norms computed at several frequency points. Fields due to *D*_1_ are clearly more dominant at point I, which is far away from the resonance frequency of *D*_2_. Surface charge distributions at the frequency points II, III, and IV (around the Fano resonance), clearly demonstrate that *D*_2_ interferes with *D*_1_. Additionally, comparison of charge distributions (and magnetic field norms) at points II and III reveals that the sharp roll-off of the transmittance between the EIT point at 8.35 THz and the graphene's dipolar SPP at 9 THz is due to the phase change of the field distribution at points II and III.

**Figure 4:**
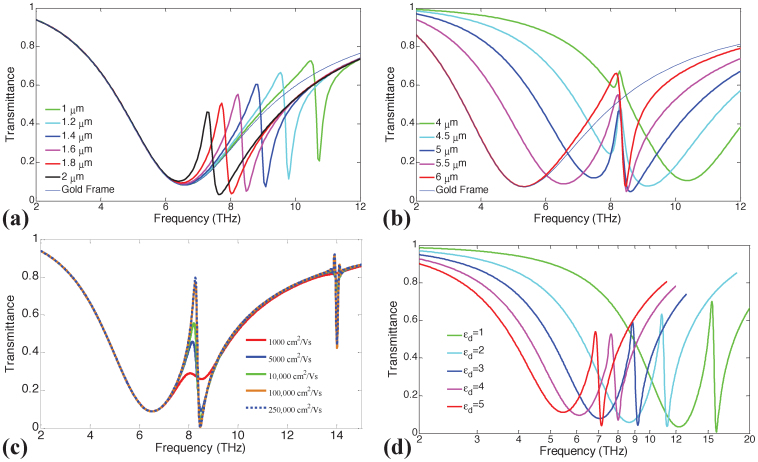
(a) Transmittance of the resonator with *S*_1_ = 5.5 μm, *ε_d_* = 3.5, *μ* = 10,000 cm^2^/Vs, and *μ_c_* = 1500 meV for various values of *S*_2_.The transmittance of only the gold frame with *S*_1_ = 5.5 μm is plotted as a reference in thin blue line. (b) Transmittance of the resonator with *S*_2_ = 1.6 μm, *ε_d_* = 3.5, *μ* = 10,000 cm^2^/Vs, and *μ_c_* = 1500 meV for various values of *S*_1_. The transmittance of only the gold frame with *S*_1_ = 6 μm is plotted as a reference in thin blue line. (c) Transmittance of the resonator with *S*_1_ = 5.5 μm, *S*_2_ = 1.6 μm, *ε_d_* = 3.5, and *μ_c_* = 1500 meV for various values of *μ*. (d) Transmittance of the resonator with *S*_1_ = 5.5 μm, *S*_2_ = 1.6 μm, *μ* = 10,000 cm^2^/Vs, and *μ_c_* = 1500 meV for various values of *ε_d_*.

The effect of geometrical dimensions on the response of the Fano resonator is characterized next. For the first set of simulations, *S*_1_ = 5.5 μm, *ε_d_* = 3.5, *μ* = 10,000 cm^2^/Vs, and *μ_c_* = 1500 meV, while *S*_2_ is varied between 1 μm and 2 μm. Transmittance of the resonator is computed for each value of *S*_2_ [Fig. 4(a)]. Increasing *S*_2_ (for example by setting it to 2 μm) red-shifts the resonance of *D*_2_ as it increases the graphene patch's effective dipole length. This moves the resonant frequencies of *D*_1_ and *D*_2_ closer to each other and forces them to couple more strongly. As a result, a higher increase in the transmittance within the EIT band [with respect to the transmittance of only *D*_1_ plotted in Fig. 4(a) as thin blue line] is observed. On the other hand, when *S*_2_ is decreased (for example by setting it to 1 μm), the resonance frequencies of *D*_1_ and *D*_2_ get sufficiently detuned. In this case, transmittance follows very closely the transmittance of only *D*_1_ with a much smaller relative increase in the EIT band. For the second set of simulations, *S*_2_ = 1.6 μm, *ε_d_* = 3.5, *μ* = 10,000 cm^2^/Vs, and *μ_c_* = 1500 meV, while *S*_1_ is varied between 4 μm and 6 μm. Transmittance of the resonator is computed for each value of *S*_1_ [Fig. 4(b)]. As expected, increasing *S*_1_ red-shifts the resonance of *D*_1_ as it increases the frame's effective dipole length. For *S*_1_ = 6 μm, a higher increase in the transmittance within the EIT band [with respect to the transmittance of only *D*_1_ plotted in Fig. 4(b) as thin blue line] is observed. For *S*_1_ between 4.5 μm and 4 μm, the asymmetric Fano line-shape appears on the left side of the resonance frequency of *D*_1_. These results clearly indicate that depending on the target application, the dimensions of the graphene patch and the gold frame can be fine-tuned. For example, if one wants to design an optical modulator using the Fano resonator proposed here, a greater modulation depth can be achieved by choosing the geometry dimensions that result in a large variation in the transmittance within the EIT band.

The effect of graphene's intrinsic loss, i.e., the value of its electron mobility, *μ*, on the response of the Fano resonator is also analyzed. For this set of simulations, *S*_1_ = 5.5 μm, *S*_2_ = 1.6 μm, *ε_d_* = 3.5, and *μ_c_* = 1500 meV, while *μ* is swept from 1,000 cm^2^/Vs to 250,000 cm^2^/Vs. Figure 4(c) shows the transmittance of the resonator computed for each value of *μ*. For lower values of *μ*, i.e., for higher loss, the absorption channel becomes dominant and the transmission efficiency is deteriorated as expected. For realistic values of *μ* around 10,000 cm^2^/Vs, the response of the Fano resonator is good; the transmittance efficiency reaches almost 60%.

Finally, the effect of the substrate on the response of the resonator is characterized. For this set of simulations, *S*_1_ = 5.5 μm, *S*_2_ = 1.6 μm, *μ* = 10,000 cm^2^/Vs, and *μ_c_* = 1500 meV, while *ε_d_* is varied between 1 (no substrate) and 5. The transmittance of the resonator is computed for each value of *ε_d_* [Fig. 4(d)]. It is clearly shown in the figure that a gradual blue-shift is observed in the response of the resonator as *ε_d_* is increased. A gradual decrease in the maximum amplitude in transmittance is also observed with an increase in *ε_d_*.

### Applications

#### Switching

The EIT window generated via the destructive interference of the dipolar SPPs induced on the gold frame and the graphene patch is tuned by controlling a gate voltage applied to the graphene patch (as discussed for example in recent studies^8^). To simulate the effect of the variation in the gate voltage, graphene's chemical potential *μ_c_* is swept between 500 meV and 1500 meV while *ε_d_* = 3.5 and *μ* = 10,000 cm^2^/Vs.

The transmittance of the structure constructed using unit cells with only the graphene patch with *S*_2_ = 1.6 μm is computed for various values of *μ_c_* [Fig. 2 (b)]. The figure clearly demonstrates that increasing *μ_c_* leads to a blue shift in the resonance frequency of *D*_2_ and comparatively stronger extinction amplitude 1 − *T* (where *T* denotes the transmittance) at the resonance frequency.

**Figure 5:**
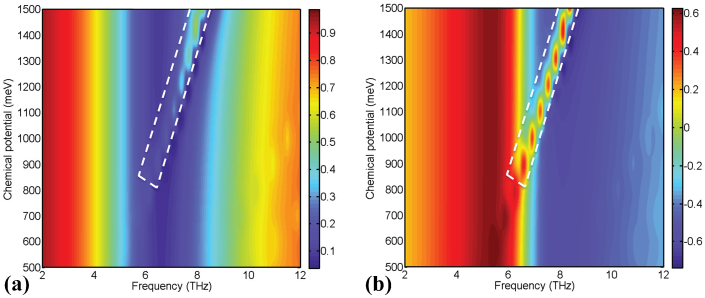
(a) Transmittance of the resonator with *S*_1_ = 5.5 μm, *S*_2_ = 1.6 μm, *ε_d_* = 3.5, and *μ* = 10,000 cm^2^/Vs for various values of *μ_c_* between 500 meV and 1500 meV.(b) Same as in (a) but for the phase of the transmission. The dashed white lines highlight the plasmonic EIT-like zone.

**Figure 6:**
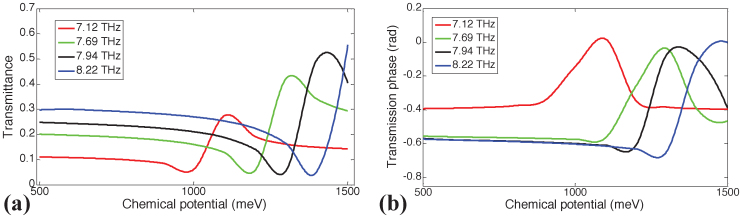
Switching applications: (a) Transmittance as function of *μ_c_* for different values of the operation frequency: 7.1, 7.7, 7.94 and 8.22 THz.*S*_1_ = 5.5 μm, *S*_2_ = 1.6 μm, *ε_d_* = 3.5, and *μ* = 10,000 cm^2^/Vs. (b) Same as in (a) but for the phase of transmission.

Similarly, transmittance and the phase of transmission of the structure constructed using unit cells with the gold frame with *S*_1_ = 5.5 μm and the graphene patch with *S*_2_ = 1.6 μm is computed for the same values of *μ_c_* [Figs. 5(a) and 5(b)]. The figures clearly show that the spectral location and the shape of the EIT window can be tuned by varying *μ_c_*. This easily tunable EIT window can be utilized as a mechanism to make switches at THz frequencies. For example, as shown in Fig. 6(a), the transmittance at 7.94 THz can be switched between 0.4% to 53% by simply varying the chemical potential *μ_c_* by an amount of Δ*μ_c_* = (1430 − 1280) meV = 150 meV. As a result a maximum modulation index of 0.52 is achieved through a small variation of Δ*μ_c_* = 150 meV. The voltage-controlled resonator has in addition the potential to be used as a phase modulation device. The highly dispersive propagation within the EIT band has a steep spectral variation in transmission phase *ϕ* [Fig. 2(c)]. This feature allows to modulate the phase of the transmitted signal to a substantial degree. As shown in Fig. 6(b), the phase of the transmission at 8.22 THz can be changed by Δ*ϕ* = 0.68 rad, by simply varying the chemical potential *μ_c_* by the same amount as for the amplitude. The amplitude and phase modulation index can be further improved by optimization of spectral positions for the interference of the resonances. For example, maximum modulation index for the amplitude can be increased to 0.65 by setting *S*_1_ = 6 μm [Fig. 4(b)]. The commercial applications for this integrated THz modulator are in the areas of communication systems, high speed Mach-Zehnder modulators, phase array antennas and time-domain spectroscopy^35^.

#### Slow light and sensing

**Figure 7:**
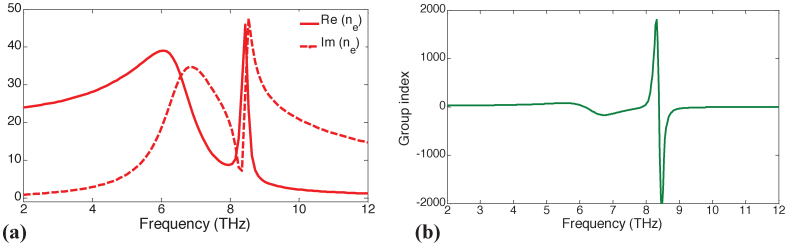
Retrieved (a) effective refractive index *n_e_* and (b) effective group index *n_g_* versus frequency. *S*_1_ = 5.5 μm, *S*_2_ = 1.6 μm, *ε_d_* = 3.5, *μ* = 10,000 cm^2^/Vs, and *μ_c_* = 1500 meV.

The effective refractive and group indices, *n_e_* and *n_g_*, of the resonator design with *S*_1_ = 5.5 μm, *S*_2_ = 1.6 μm, *ε_d_* = 3.5, *μ* = 10,000 cm^2^/Vs, and *μ_c_* = 1500 meV are extracted. To this end, a homogenous slab with refractive index *n_e_*, which generates the same S-parameters as the proposed design, is found using the retrieval method described in reference^36^. Then, the effective group index is computed using the relation *n_g_*(*ω*) = *n_e_*(*ω*) + *ω∂n_e_*(*ω*)/*∂ω*^36^. The retrieved *n_e_* and *n_g_* are shown in Figs. 7(a) and 7(b), respectively; the value of *n_g_* exceeds 1,400 within the EIT window. This value is much higher than that of the other plasmonic resonators solely made of metals^2^. High values of *n_g_* clearly demonstrate that the proposed Fano resonator design has the potential to be used in slow light applications including ultra-sensitive biomolecule detection.

## Discussion

To the best of our knowledge, our work is the first to report on EIT generated at THz frequencies using a hybrid graphene-gold structure. This novel design makes use of the fact that dipolar SPP induced on graphene are much narrower than those induced on gold to induce the asymmetric Fano resonance shape in the spectrum. This concept is novel and has not been exploited before. The use of this idea equipped the proposed Fano resonator with the following properties that are superior to “classical” Fano resonators constructed using only noble metals:

1. *Polarization Independence:* The design is polarization independent since it does not require excitation of originally dark modes via symmetry breaking.
2. *Dynamic Tunability:* The spectral location and shape of the Fano resonance (and the EIT window) can be tuned by varying the chemical potential of the graphene patch. This can be dynamically achieved by applying a gate voltage to the graphene patch.
3. *Higher Group Index:* Group index extracted around the EIT window is higher than that reported in literature previously. This equips our design with a high sensitivity to the changes in the background medium's refractive index.

It should be emphasized here that because gold's intrinsic loss is higher than graphene's, one would expect the overall loss in the hybrid resonator would increase destroying the EIT. But it is observed that within the EIT region, where the destructive interference occurs, the radiation losses are suppressed. This is indeed why adding the gold frame to the system decreases the total losses within the EIT region instead of increasing them. The high transmittance values, which reach 80% as could be seen in Fig. 4(c), fully demonstrate this fact.

The proposed resonator has potential applications in designing efficient switches and ultra-sensitive bio-detectors that can be operated at THz frequencies. Our work also demonstrates the possibility of these via numerical simulations with realistic parameters.

## Methods

### Material models

The complex dielectric constant of gold is accurately modeled at THz frequencies using the Drude model with plasma frequency *ω_p_* = 1.37 × 10^16^ rad/s and damping constant *γ_c_* = 39.47 × 10^12^ rad/s^34^. The complex surface conductivity *σ*_S,G_ for a graphene layer is calculated from Kubo's formula^8^,^14^: 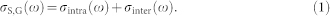
Here, *σ*_intra_ and *σ*_inter_ represent the intra- and inter-band transitions in the graphene layer and their expressions could be found in many recent studies^19^,^20^. At low THz frequencies, where we have *σ*_intra_ ≫ *σ*_inter_, *σ*_S,G_ could be approximated by a Drude model: 
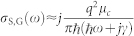
, where *ω* is the angular frequency, *q* is the electron charge, *ℏ* is the reduced Planck constant, *μ_c_* is the chemical potential of the doped graphene layer, and *γ* is the damping constant. In this work, it is assumed that the thickness of the graphene layer *δ* = 1 nm; this choice was mainly motivated and justified by the seminal work of Vakil and Engheta^19^. Since *δ* is much smaller than the wavelength at THz frequencies, graphene's bulk conductivity can be simplified as *σ*_V,G_ = *σ*_S,G_/*δ*. Using Ampere's law in stationary regime and Ohm's law, one can calculate complex relative bulk permittivity of graphene as 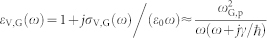 with the plasma frequency of graphene 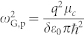. The damping constant used for graphene is given by *γ* = −(*eℏv_f_*^2^)/(*μμ_c_*), where *v_f_* = *c*/300 m/s is the Fermi velocity and *μ* is the electron mobility. This expression clearly demonstrates the dependence of graphene layer's complex permittivity *ε*_V,G_ on the chemical potential *μ_c_* and operating frequency *ω*. This dependence suggests that *μ_c_* can be varied to tune the spectral location and line-shape of the Fano resonance.

These material models are used in the finite element program COMSOL Multiphysics to carry out the simulations of electromagnetic wave interactions on the design presented in Figs. 1(a), 1(b), and 1(c). The results of these simulations are presented in the previous sections. These simulation results can also be “predicted” by the response of an RLC circuit as described in the next section.

### RLC analytic model

**Figure 8:**
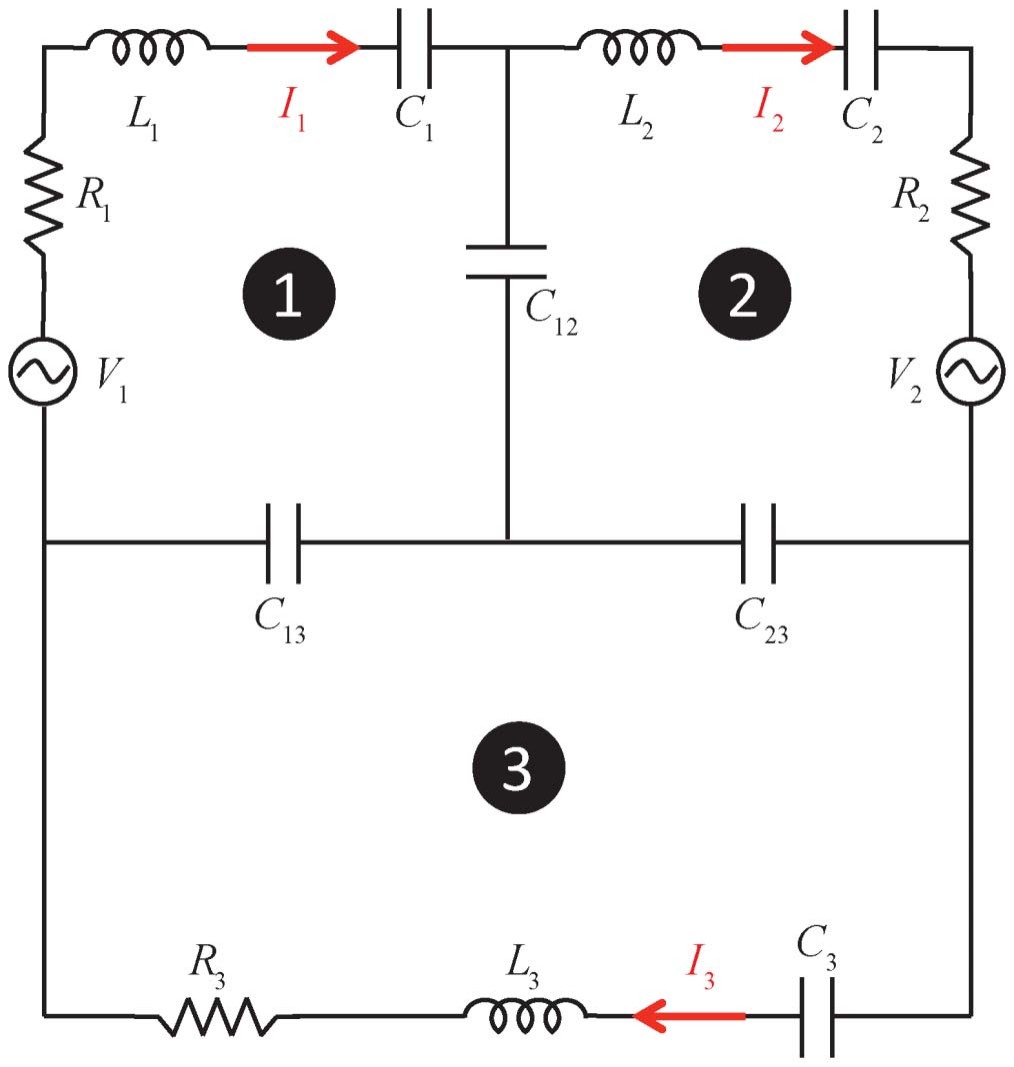
Schematics of the three-loop RLC circuit. Two-loop circuit is obtained by removing the third loop by short-circuiting *C*_13_ and *C*_23_.

The optical response of the proposed Fano resonator can be mathematically modeled using coupled oscillator equations. In this work, an RLC circuit model is used to replicate the optical response of the Fano resonator^37^. In the circuit model, each of the dipolar SPPs induced on the gold frame and the graphene patch are represented by an RLC loop as shown in Fig. 8. Note that the additional third RLC loop in the circuit is needed to take into account the coupling from higher order SPPs induced on the gold frame and graphene patch beyond 12 THz. The loops are connected through three capacitors *C*_12_, *C*_13_, and *C*_23_, which model the coupling between the different SPPs. This RLC circuit can be mathematically represented as a system of three coupled equations: 
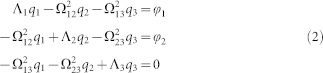
Here, *q_i_*, *i* = 1,2,3, is the charge due to the steady state current *I_i_* = *jωq_i_* flowing in the loop indexed with *i*. The term Ω*_ij_* = (*L_i_C_ij_*)^−0.5^, *i*,*j* = 1,2,3, *i* < *j*, couples the three equations to each other, 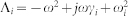 represents the “self-coupling”, where *γ_i_* = *R_i_*/*L_i_* is the damping coefficient, *ω_i_* = (*L_i_C_xi_*)^−0.5^, is the LC resonant frequency of the loop indexed with *i*, and 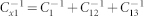, 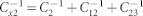, and 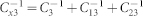. The terms *φ_i_* = *V_i_*/*L_i_*, *i* = 1,2, on the right side of the system of equations (2) represent the excitation, i.e., the direct coupling from the incident field to the SPPs. The time average power drawn by the circuit is *P* = 1/2Re[*V*_1_*I*_1_* + *V*_2_*I*_2_*] and this quantity should be equivalent to the extinction coefficient 1 − *T* of the Fano resonator. By minimizing the difference between *P* and 1 − *T* numerically, *ω_i_*, *γ_i_*, *φ_i_*, and Ω*_ij_* can be found. Once these coefficients are known, one can easily obtain the physical parameters, *R_i_*, *L_i_*, *C_i_*, *i* = 1,2,3 and *C*_12_, *C*_13_, and *C*_23_, which describe the RLC circuit.

The mathematical model described by the system of equations (2) provides additional physical insight into the response of the Fano resonator. For example, *φ_i_*, *i* = 1,2, represent the amount of relative power each resonant mode receives from the incident electromagnetic field. Non-zero values of *φ_i_* indicate that both modes have dipole moments along the polarization vector of the incident field and they can be directly excited. Variables Ω_12_, Ω_13_, and Ω_23_ represent the amount of (energy) coupling between the modes. For example a high Ω_12_ means that the coupling between *D*_1_ and *D*_2_ is strong, which indicates high transmittance *T* within the EIT region. This is demonstrated by an example as described next.

Parameters of the circuit with three loops is obtained by minimizing the difference between *P* and 1 − *T* of the Fano resonator with *S*_1_ = 5.5 μm, *S*_2_ = 1.6 μm, *ε_d_* = 3.5, and *μ* = 10,000 cm^2^/Vs for various values of *μ_c_* between 500 meV and 1500 meV. The values of the extracted parameters are provided for each value of *μ_c_* in Table I. The effect of the increase in *μ_c_* can be seen with a blue shift in the resonance frequency *ω*_2_ that corresponds to *D*_2_. Additionally, a consistent increase in Ω_12_, which represents the strength of coupling between *D*_1_ and *D*_2_, is observed. As mentioned above, the increase in coupling strength, i.e., increase in Ω_12_ translates as increased *T* within the EIT region [see Fig. 5(a)]. Note that as expected, increasing *μ_c_* does not effect characteristics of *D*_1_. As shown in Table I, the resonant and damping frequencies, *ω*_1_ and *γ*_1_, corresponding to *D*_1_ remain unchanged.

## Author Contributions

M.A. and M.F. carried out the simulations and prepared the figures. HB supervised the research. All authors contributed equally to writing the main manuscript.

## Figures and Tables

**Table 1:** Parameters of the RLC circuit model representing the Fano resonator with *S*_1_ = 5.5 μm, *S*_2_ = 1.6 μm, *ε_d_* = 3.5, and *μ* = 10,000 cm^2^/Vs for various values of *μ_c_* between 500 meV and 1500 meV. Units of *ω*_1_, *ω*_2_, *ω*_3_, Ω_12_, Ω_13_, and Ω_23_ are (meV) while the units of *φ*_1_ and *φ*_2_ are (mV/H)

μ_c_	ω_1_	ω_2_	ω_3_	Ω_12_	Ω_13_	Ω_23_	γ_1_	γ_2_	γ_3_	*φ*_1_	*φ*_2_
500	31.5	20.8	78.5	0.10	36.89	0.10	14.34	1.07	121.95	191.54	3.53
600	31.5	22.1	78.2	0.10	36.78	7.66	14.34	1.38	122.05	191.64	0.10
700	31.5	23.9	75.5	1.20	36.05	9.67	14.34	1.18	114.32	191.87	0.13
800	31.5	25.5	78.6	1.28	36.78	11.74	14.34	0.89	125.99	191.91	2.34
900	31.5	27.0	76.1	1.90	36.04	13.65	14.34	0.81	118.62	192.28	3.80
1000	31.5	28.5	74.4	1.92	35.43	16.38	14.34	0.66	114.24	192.57	6.40
1100	31.5	29.7	75.9	6.28	35.86	12.42	14.34	0.77	120.62	192.92	0.10
1200	31.5	30.9	77.7	6.97	36.14	15.17	14.34	0.69	129.68	193.02	1.97
1300	31.5	32.1	74.2	7.84	35.01	16.95	14.34	0.59	120.52	193.25	4.07
1400	31.5	33.4	68.1	8.13	32.62	22.32	14.34	0.06	106.08	192.73	14.65
1500	31.5	34.3	64.5	9.83	31.45	21.51	14.34	0.06	98.05	192.90	13.91

## References

[b1] Miroshnichenko A. E., Flach S. & Kivshar Y.-S. Fano resonances in nanoscale structures. Rev. Mod. Phys. 82, 2257–2298 (2010).

[b2] Chiam S.-Y., Singh R., Rockstuhl C., Lederer F., Zhang W. & Bettiol A. A. Analogue of electromagnetically induced transparency in a terahertz metamaterial. Phys. Rev. B 80, 153103 (2009).

[b3] Luk'yanchuk B., Zheludev N., Maier S., Halas N., Nordlander P., Giessen H. & Chong C. The Fano resonance in plasmonic nanostructures and metamaterials. Nat. Mater. 9, 707–715 (2010). 20733610 10.1038/nmat2810

[b4] Mukherjee S., Sobhani H., Lassiter J. B., Bardhan R., Nordlander P. & Halas N. J. Fanoshells: nanoparticles with built-in Fano resonance. Nano Lett. 10, 2694–2701 (2010). 20509616 10.1021/nl1016392

[b5] Rahmani M., Luk'yanchuk B. & Hong M. Fano resonance in novel plasmonic nanostructures. Laser Photon. Rev. 7, 329–349 (2013).

[b6] Wu C., Khanikaev A. B., Adato R., Arju N., Yanik A. A., Altuğ H. & Shvets G. Fano-resonant asymmetric metamaterials for ultrasensitive spectroscopy and identification of molecular monolayers. Nat. Mater. 11, 69–75 (2012). 22081082 10.1038/nmat3161

[b7] Chung T., Lee S.-Y., Song E. Y., Chun H. & Lee B. Plasmonic nanostructures for nanoscale bio-sensing. Sensors 11, 10907–10929 (2011). 22346679 10.3390/s111110907 PMC3274321

[b8] Lee S. H., Choi M., Kim T. T., Lee S., Liu M., Yin X., Choi H. K., Lee S. S., Choi C. G., Choi S. Y., Zhang X. & Min B. Switching terahertz waves with gate-controlled active graphene metamaterials. Nat. Mater. 11, 936–941 (2012). 23023552 10.1038/nmat3433

[b9] Novoselov K. S., Geim A. K., Morozov S. V., Jiang D., Zhang Y., Dubonos S. V., Grig-orieva I. V. & Firsov A. A. Electric field effect in atomically thin carbon films. Science 306, 666–669 (2004). 15499015 10.1126/science.1102896

[b10] Castro Neto A. H., Guinea F., Peres N. M. R., Novoselov K. S. & Geim A. K. The electronic properties of graphene. Rev. Mod. Phys. 81, 109–162 (2009).

[b11] Geim A. K. & Novoselov K. S. The rise of graphene. Nat. Mater. 6, 183–191 (2007). 17330084 10.1038/nmat1849

[b12] Nair R. R., Blake P., Grigorenko A. N., Novoselov K. S., Booth T. J., Stauber T., Peres N. M. R. & Geim A. K. Fine structure constant defines visual transparency of graphene. Science 320, 1308 (2008). 18388259 10.1126/science.1156965

[b13] Xia F. N., Mueller T., Lin Y. M., Valdes-Garcia A. & Avouris P. Ultrafast graphene photodetector. Nat. Nanotechnol. 4, 839–843 (2009). 19893532 10.1038/nnano.2009.292

[b14] Hanson G. W. Dyadic Green's functions and guided surface waves for a surface conductivity model of graphene. J. Appl. Phys. 103, 064302 (2006).

[b15] Hwang E. H. & Das Sarma S. Dielectric function, screening, and plasmons in two-dimensional graphene. Phys. Rev. B 75, 205418 (2007).

[b16] Rana F. Graphene terahertz plasmon oscillators. IEEE Trans. Nanotechnol. 7, 91–99 (2008).

[b17] Jablan M., Buljan H. & Soljacic M. Plasmonics in graphene at infrared frequencies. Phys. Rev. B 80, 245435 (2009).

[b18] Novoselov K. S., Falko V. I., Colombo L., Gellert P. R., Schwab M. G. & Kim K. A roadmap for graphene. Nature 490, 192–200 (2012). 23060189 10.1038/nature11458

[b19] Vakil A. & Engheta N. Transformation optics using graphene. Science 332, 1291–1294 (2011). 21659598 10.1126/science.1202691

[b20] Koppens F. H. L., Chang D. E. & de Abajo F. J. G. Graphene plasmonics: A platform for strong light-matter interactions. Nano Lett. 11, 3370–3377 (2011). 21766812 10.1021/nl201771h

[b21] Christensen J., Manjavacas A., Thongrattanasiri S., Koppens F. H. L. & de Abajo F. J. G. Graphene plasmon waveguiding and hybridization in individual and paired nanoribbons. ACS Nano 6, 431–440 (2012). 22147667 10.1021/nn2037626

[b22] Locatelli A., Capobianco A. D., Midrio M., Boscolo S. & De Angelis C. Graphene-assisted control of coupling between optical waveguides. Opt. Express 20, 28479–28484 (2012). 23263083 10.1364/OE.20.028479

[b23] Shin K.-Y., Hong J.-Y. & Jang J. Micropatterning of graphene sheets by inkjet printing and its wideband dipole-antenna application. Adv. Mater. 23, 2113–2118 (2011). 21484894 10.1002/adma.201100345

[b24] Tamagnone M., Gómez-Díaz J. S., Mosig J. R. & Perruisseau-Carrier J. Reconfigurable terahertz plasmonic antenna concept using a graphene stack. Appl. Phys. Lett. 101, 214102 (2012).

[b25] Filter R., Farhat M., Steglich M., Alaee R., Rockstuhl C. & Lederer F. Tunable graphene antennas for selective enhancement of THz-emission. Opt. Express 21, 3737–3745 (2013). 23481830 10.1364/OE.21.003737

[b26] Thongrattanasiri S., Koppens F. H. L. & de Abajo F. J. G. Complete optical absorption in periodically patterned graphene. Phys. Rev. Lett. 108, 047401 (2012). 22400887 10.1103/PhysRevLett.108.047401

[b27] Alaee R., Farhat M., Rockstuhl C. & Lederer F. A perfect absorber made of a graphene micro-ribbon metamaterial. Opt. Express 20, 28017–28024 (2012). 23263036 10.1364/OE.20.028017

[b28] Vakil A. & Engheta N. Fourier optics on graphene. Phys. Rev. B 85, 075434 (2012).

[b29] Chen P.-Y. & Alù A. Atomically thin surface cloak using graphene monolayers. ACS Nano 5, 5855–5863 (2011). 21662981 10.1021/nn201622e

[b30] Farhat M., Rockstuhl C. & Bağcı H. A 3D tunable and multi-frequency graphene plasmonic cloak. Opt. Express 21, 12592–12603 (2013). 23736478 10.1364/OE.21.012592

[b31] Gan X., Mak K. F., Gao Y., You Y., Hatami F., Hone J., Heinz T. F. & Englund D. Strong enhancement of light-matter interaction in graphene coupled to a photonic crystal nanocavity. Nano Lett. 12, 5626–5631 (2012). 23043452 10.1021/nl302746n

[b32] Yan R., Sensale-Rodriguez B., Liu L., Jena D. & Xing H. G. A new class of electrically tunable metamaterial terahertz modulators. Opt. Express 20, 28664–28671 (2012). 23263104 10.1364/OE.20.028664

[b33] Lv R., Li Q., Botello-Mendez A. R., Hayashi T., Wang B., Berkdemir A., Hao Q., Elias A. L., Cruz-Silva R., Gutierrez H. R., Kim Y. A., Muramatsu H., Zhu J., Endo M., Terrones H., Charlier J.-C., Pan M. & Terrones M. Nitrogen-doped graphene: beyond single substitution and enhanced molecular sensing. Sc. Rep. 10.1038/srep00586 (2012). 10.1038/srep00586 PMC3421434 22905317

[b34] Ordal, M. A. Long L. L. Bell R. J. Bell S. E. Bell R. R. Alexander R. W. & Ward C. A. “Optical properties of the metals Al, Co, Cu, Au, Fe, Pb, Ni, Pd, Pt, Ag, Ti, and W in the infrared and far infrared. Appl. Opt. 22, 1099–1119 (1983). 18195926 10.1364/ao.22.001099

[b35] Rahm M., Li J.-S. & Padilla W. J. THz wave modulators: a brief review on different modulation techniques. J. Infrared Millim. Terahertz Waves 34, 1–27 (2012).

[b36] Smith D. R., Vier D. C., Koschny T. & Soukoulis C. M. Electromagnetic parameter retrieval from inhomogeneous metamaterials. Phys. Rev. E 71, 036617 (2005). 10.1103/PhysRevE.71.036617 15903615

[b37] Amin M. & Bağcı H. Investigation of Fano resonances induced by higher order plasmons modes on a circular disk with an elongated cavity. Prog. Electromag. Res. 130, 187–206 (2012).

